# Occurrence of invasive pneumococcal disease and number of excess cases due to influenza

**DOI:** 10.1186/1471-2334-6-58

**Published:** 2006-03-20

**Authors:** Katarzyna Grabowska, Liselotte Högberg, Pasi Penttinen, Åke Svensson, Karl Ekdahl

**Affiliations:** 1 Department of Epidemiology, Swedish Institute for Infectious Disease Control (EPI/SMI), SE-171 82 Solna, Sweden; 2 Department of Medical Epidemiology and Biostatistics (MEB), Karolinska Institutet, SE-171 77 Stockholm, Sweden; 3 Stockholm Group for Epidemic Modelling (S-GEM), Stockholm, Sweden; 4 European Centre for Disease Prevention and Control (ECDC), SE-171 83 Stockholm, Sweden

## Abstract

**Background:**

Influenza is characterized by seasonal outbreaks, often with a high rate of morbidity and mortality. It is also known to be a cause of significant amount secondary bacterial infections. *Streptococcus pneumoniae *is the main pathogen causing secondary bacterial pneumonia after influenza and subsequently, influenza could participate in acquiring Invasive Pneumococcal Disease (IPD).

**Methods:**

In this study, we aim to investigate the relation between influenza and IPD by estimating the yearly excess of IPD cases due to influenza. For this purpose, we use influenza periods as an indicator for influenza activity as a risk factor in subsequent analysis. The statistical modeling has been made in two modes. First, we constructed two negative binomial regression models. For each model, we estimated the contribution of influenza in the models, and calculated number of excess number of IPD cases. Also, for each model, we investigated several lag time periods between influenza and IPD. Secondly, we constructed an "influenza free" baseline, and calculated differences in IPD data (observed cases) and baseline (expected cases), in order to estimate a yearly additional number of IPD cases due to influenza. Both modes were calculated using zero to four weeks lag time.

**Results:**

The analysis shows a yearly increase of 72–118 IPD cases due to influenza, which corresponds to 6–10% per year or 12–20% per influenza season. Also, a lag time of one to three weeks appears to be of significant importance in the relation between IPD and influenza.

**Conclusion:**

This epidemiological study confirms the association between influenza and IPD. Furthermore, negative binomial regression models can be used to calculate number of excess cases of IPD, related to influenza.

## Background

Influenza, with its annual epidemics, is the infection associated with highest mortality in the developed world. In the United States, an average of more than 18,000 annual deaths are due to influenza, and the influenza epidemics cause almost 50,000 annual hospitalizations in influenza and pneumonia among elderly people [1].

*Streptococcus pneumoniae *is the most important pathogen causing secondary bacterial pneumonia after influenza [2-4]. The influenza virus infection, makes the respiratory epithelial cells more susceptible to pneumococcal invasion [5], and in mouse models, the increased susceptibility to secondary bacterial pneumonia after influenza has in part shown to be caused by excessive IL-10 production and reduced neutrophil function in the lungs. In several early, hospital-based studies, pneumococcal pneumonia has been shown to be a complication of clinical diagnosis of influenza [6-9]. In spite of modern intensive care, Invasive pneumococcal disease (IPD) is associated with a high case fatality rate [10,11]. Known risk factors for IPD are age (the very old or the very young), male sex, and underlying debilitating conditions[12]. In Sweden, the overall yearly incidence of IPD is 15 per 100,000, and in the above 65 years age group the incidence may be as high as 40 to 50 per 100,000 [10]. In the United States, IPD is estimated to cause more than 3,400 deaths per year among elderly people [3]. Older generation pneumococcal polysaccharide vaccines have proven to be effective against IPD in adults, and newer conjugate pneumococcal vaccine also are effective in young children [13,14]. In elderly persons and other medical risk groups, influenza and pneumococcal vaccines are commonly used together [15].

Both IPD and influenza have distinct seasonal patterns, with winter peaks [16-18]. Besides this seasonal pattern, there are year-to-year variations both in intensity and timing of occurrence [18-20]. Schwartzman et al were the first to document a temporal association between influenza and IPD in the early 1970s [21]. The same finding has later been described in studies from Scotland [22], the Netherlands [23], and the United States [4,24,25].

The aim of this studies it to investigate the association between influenza and IPD using Swedish surveillance data. Poisson regression has often been used in epidemiological studies when an outcome variable (e.g. number of IPD cases) is a rare occurrence, and Poisson regression has recently been applied in studies investigating the association between influenza and mortality [26,27]. However, one of the main characteristics of the Poisson model is that its variance equals its mean. In other words, if a Poisson model is fitted to data with a variance greater than its mean (overdispersion), the variance will be underestimated. To overcome this limitation, we apply negative binomial models, which give the same estimation of a mean value as a Poisson model, but include overdispersion in the variance [28].

## Methods

### IPD data

Since 1994, all invasive pneumococcal isolates obtained in Sweden (one per patient) have been reported to the Swedish Institute for Infectious Diseases Control (SMI). An isolate is defined as invasive if it is retrieved from blood, cerebrospinal fluid or other normally sterile sites. All such isolates, reported to the SMI from 1 January 1994 to 28 March 2004 (n = 12,010) were included in the study. Most isolates (95%) were collected from blood, followed by cerebrospinal fluid (5%); and other normally sterile sites (less than 1%). Fifty percent of the isolates were from elderly persons (65 years of age of older), 43% were from adults (20–64 years of age), and 7% of the isolates were from children and teenagers. In the analysis, the date of culture was used. Since this date was not always specified in the reports, the samples where this date is lacking were omitted (n = 373).

The symptoms of invasive disease are of acute nature; the time of onset of disease and the date of culture is assumed to coincide within 1 to 2 days. The data used in the analysis is aggregated to weeks, which should reflect the correct week of onset of disease. Finally, as mentioned above, invasive disease is often of sudden onset. This promote rapid culturing and also diagnosing of the IPD, therefore the assumption was made, that the vast majority of the invasive cases of pneumococcal infections are diagnosed correctly.

### Influenza data

In Sweden, the influenza surveillance is based on weekly reports (Monday to Sunday of each individual week) on the number of influenza cases diagnosed by antigen detection, nucleic acid amplification and/or virus isolation by the local laboratories to SMI. Serology reports are not included in these reports. From 1994 to 2001, the registration of influenza occurred from week 43 to week 16. From 2001/2002, the influenza-reporting season was extended from week 40 to 20. For the influenza reports, we used the same time period (January 1994 to March 2004) as for the IPD isolates (n = 10,498). The age distribution of influenza cases varied between individual years. Data on age of influenza cases is available from the season 1998/1999, and shows that an average of 47% of the cases were elderly (above 65 years) and 39% adults (20–64 years) during the period 1998–2004.

We defined influenza activity as presence of laboratory reports of influenza cases. As the laboratory cases mainly originate from patients with severe disease in need of hospital care, this data might not reflect the true influenza activity among the general population. Since the season 1999/2000 additional components have been added to the Swedish influenza surveillance system, based on reports of the number of patients with influenza-like illness from about 120 sentinel physicians in outpatient care. During the time when both systems have been running in parallel, the time periods for influenza activity have coincided [29], supporting the hypothesis, that the laboratory surveillance system adequately mirrors community influenza activity despite the selected population it is based on. This consistency does also indicate that even though the criteria for culturing might have changed during the observation period, it has not affected the definition of the annual influenza period by the laboratory reporting.

As mentioned above, the influenza data is based on laboratory reports, and mainly reflects the hospitalized patients and not the population as total. Hence, the amount of cases may not be representative for the total population. However, the time of occurrence coincide in the sentinel system and the laboratory reports. In order to incorporate the time of occurrence but not the total amount of influenza (according to laboratory reports) in the models, an indicator for influenza is used (one if influenza is present and zero if absent).

### Models

In subsequent models, following variables and abbreviations are used:

β_inf _Parameter for the effect of presence of influenza

β_int _Parameter for the intercept

β_sin _and β_cos _estimated cyclic parameters

β_year _Estimated yearly trend

μ_i _Modeled mean value, with the Gamma distribution, Γ(α_*i*_,δ)

*x*_0*i *_Denotes the index variables for influenza (1 if influenza is present, 0 if absent)

*x*_1*i *_Indicates the year

*i *is an index variable form 1 to 533 for the models

*j *is an index variable from 1 to 52 for the baseline

To evaluate the relation between influenza and IPD, two negative binomial regression models were constructed. The question of interest was if the presence of influenza affects the number of IPD cases. The models needed to capture the seasonal characteristics in data, hence the models include seasonal terms: sin(2π52ti)
 MathType@MTEF@5@5@+=feaafiart1ev1aaatCvAUfKttLearuWrP9MDH5MBPbIqV92AaeXatLxBI9gBaebbnrfifHhDYfgasaacH8akY=wiFfYdH8Gipec8Eeeu0xXdbba9frFj0=OqFfea0dXdd9vqai=hGuQ8kuc9pgc9s8qqaq=dirpe0xb9q8qiLsFr0=vr0=vr0dc8meaabaqaciaacaGaaeqabaqabeGadaaakeaacqqGZbWCcqqGPbqAcqqGUbGBdaqadaqaamaalaaabaGaeGOmaiJaeqiWdahabaGaeGynauJaeGOmaidaaiabdsha0naaBaaaleaacqWGPbqAaeqaaaGccaGLOaGaayzkaaaaaa@3A09@ and cos(2π52ti)
 MathType@MTEF@5@5@+=feaafiart1ev1aaatCvAUfKttLearuWrP9MDH5MBPbIqV92AaeXatLxBI9gBaebbnrfifHhDYfgasaacH8akY=wiFfYdH8Gipec8Eeeu0xXdbba9frFj0=OqFfea0dXdd9vqai=hGuQ8kuc9pgc9s8qqaq=dirpe0xb9q8qiLsFr0=vr0=vr0dc8meaabaqaciaacaGaaeqabaqabeGadaaakeaacqqGJbWycqqGVbWBcqqGZbWCdaqadaqaamaalaaabaGaeGOmaiJaeqiWdahabaGaeGynauJaeGOmaidaaiabdsha0naaBaaaleaacqWGPbqAaeqaaaGccaGLOaGaayzkaaaaaa@39FF@. Additionally, the models needed to include a parameter that represents the influenza and a yearly trend. The outcome variable is the mean value of number of IPD cases μ_*i*_, with a Gamma distribution Γ(α_*i*_,δ), hence models include parameters α_*i *_and δ. In the models, each individual week, *t*_*i *_(*t*_*i *_= 1,...,52) is modeled. For analytical details, see appendix A.

Secondly, a mean baseline of all years was constructed. The baseline included the same parameters as the models, except the influenza parameter and the yearly trend. To estimate the mean baseline we used a subset of IPD data. In this subset only weeks when influenza was absent were included, in the time periods when influenza was monitored (winter season). (Hence, this subset only contained IPD data when influenza was not present.)

Since it is a mean, it includes only one season, (*j *= 1 to 52).

### Model 1

The first model was defined as:

log⁡(αi)=log⁡(δ)+βsin⁡sin⁡(2π52ti)+βcos⁡cos⁡(2π52ti)+βint⁡+βinf⁡x0i+βyearx1i
 MathType@MTEF@5@5@+=feaafiart1ev1aaatCvAUfKttLearuWrP9MDH5MBPbIqV92AaeXatLxBI9gBaebbnrfifHhDYfgasaacH8akY=wiFfYdH8Gipec8Eeeu0xXdbba9frFj0=OqFfea0dXdd9vqai=hGuQ8kuc9pgc9s8qqaq=dirpe0xb9q8qiLsFr0=vr0=vr0dc8meaabaqaciaacaGaaeqabaqabeGadaaakeaacyGGSbaBcqGGVbWBcqGGNbWzcqGGOaakcqaHXoqydaWgaaWcbaGaemyAaKgabeaakiabcMcaPiabg2da9iGbcYgaSjabc+gaVjabcEgaNjabcIcaOiabes7aKjabcMcaPiabgUcaRiabek7aInaaBaaaleaacyGGZbWCcqGGPbqAcqGGUbGBaeqaaOGagi4CamNaeiyAaKMaeiOBa42aaeWaaeaadaWcaaqaaiabikdaYiabec8aWbqaaiabiwda1iabikdaYaaacqWG0baDdaWgaaWcbaGaemyAaKgabeaaaOGaayjkaiaawMcaaiabgUcaRiabek7aInaaBaaaleaacyGGJbWycqGGVbWBcqGGZbWCaeqaaOGagi4yamMaei4Ba8Maei4Cam3aaeWaaeaadaWcaaqaaiabikdaYiabec8aWbqaaiabiwda1iabikdaYaaacqWG0baDdaWgaaWcbaGaemyAaKgabeaaaOGaayjkaiaawMcaaiabgUcaRiabek7aInaaBaaaleaacyGGPbqAcqGGUbGBcqGG0baDaeqaaOGaey4kaSIaeqOSdi2aaSbaaSqaaiGbcMgaPjabc6gaUjabcAgaMbqabaGccqWG4baEdaWgaaWcbaGaeGimaaJaemyAaKgabeaakiabgUcaRiabek7aInaaBaaaleaacqqG5bqEcqqGLbqzcqqGHbqycqqGYbGCaeqaaOGaemiEaG3aaSbaaSqaaiabigdaXiabdMgaPbqabaaaaa@847A@

In this model, a global parameter for the year was used. In other words, the same height with the same amplitude was set for each year as a mean yearly trend for all years. Hence, the influenza data indicator and the yearly trend are the sole cause of the differences between the individual years.

### Model 2

**Figure 1:**
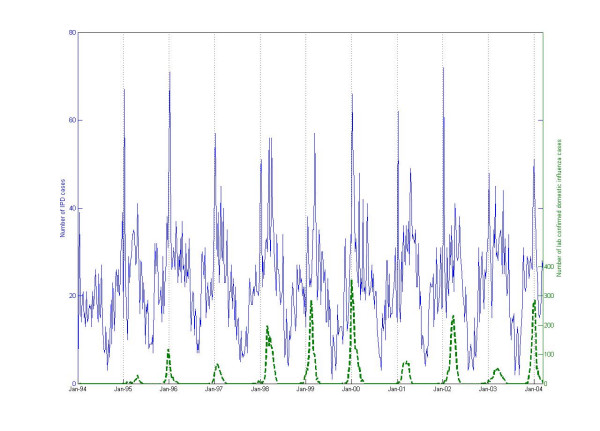
Total number of IPD diagnosis (solid line) and number of laboratory confirmed cases of influenza (dashed line).

**Figure 2:**
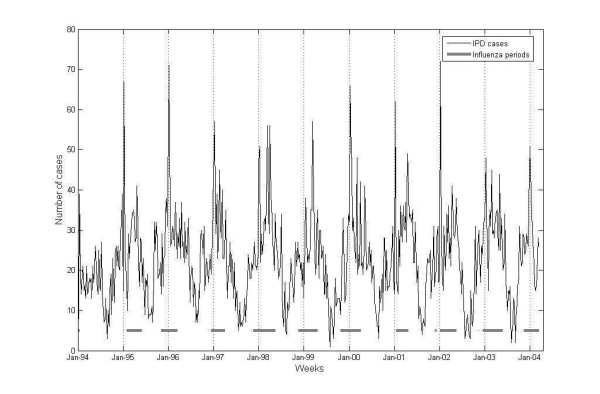
Total number of IPD diagnosis. The presence of influenza during different years is shown as horizontal solid lines.

When the IPD data was studied, it was notable that the peaks were of varying height, possibly due to variation between individual years, see Figure 1 and 2. A way of incorporating this into a model was to allow each periodic cycle to vary in height of amplitude for each year. This can be obtained by using an index variable for each year. Subsequently, in Model 2, the parameter β_year_, divided into 11 parameters (one for each year) was included. The model was defined as:

log⁡(αi)=log⁡(δ)+βsinsin⁡(2π52ti)+βcoscos⁡(2π52ti)+βint⁡+βinf⁡x0i++βyear1x1i+…+βyear11x11i
 MathType@MTEF@5@5@+=feaafiart1ev1aaatCvAUfKttLearuWrP9MDH5MBPbIqV92AaeXatLxBI9gBaebbnrfifHhDYfgasaacH8akY=wiFfYdH8Gipec8Eeeu0xXdbba9frFj0=OqFfea0dXdd9vqai=hGuQ8kuc9pgc9s8qqaq=dirpe0xb9q8qiLsFr0=vr0=vr0dc8meaabaqaciaacaGaaeqabaqabeGadaaakqaabeqaaiGbcYgaSjabc+gaVjabcEgaNjabcIcaOiabeg7aHnaaBaaaleaacqWGPbqAaeqaaOGaeiykaKIaeyypa0JagiiBaWMaei4Ba8Maei4zaCMaeiikaGIaeqiTdqMaeiykaKIaey4kaSIaeqOSdi2aaSbaaSqaaiabbohaZjabbMgaPjabb6gaUbqabaGccyGGZbWCcqGGPbqAcqGGUbGBdaqadaqaamaalaaabaGaeGOmaiJaeqiWdahabaGaeGynauJaeGOmaidaaiabdsha0naaBaaaleaacqWGPbqAaeqaaaGccaGLOaGaayzkaaGaey4kaSIaeqOSdi2aaSbaaSqaaiabbogaJjabb+gaVjabbohaZbqabaGccyGGJbWycqGGVbWBcqGGZbWCdaqadaqaamaalaaabaGaeGOmaiJaeqiWdahabaGaeGynauJaeGOmaidaaiabdsha0naaBaaaleaacqWGPbqAaeqaaaGccaGLOaGaayzkaaGaey4kaSIaeqOSdi2aaSbaaSqaaiGbcMgaPjabc6gaUjabcsha0bqabaGccqGHRaWkcqaHYoGydaWgaaWcbaGagiyAaKMaeiOBa4MaeiOzaygabeaakiabdIha4naaBaaaleaacqaIWaamcqWGPbqAaeqaaOGaey4kaScabaGaey4kaSIaeqOSdi2aaSbaaSqaaiabbMha5jabbwgaLjabbggaHjabbkhaYjabbgdaXaqabaGccqWG4baEdaWgaaWcbaGaeGymaeJaemyAaKgabeaakiabgUcaRiablAciljabgUcaRiabek7aInaaBaaaleaacqqG5bqEcqqGLbqzcqqGHbqycqqGYbGCcqqGXaqmcqqGXaqmaeqaaOGaemiEaG3aaSbaaSqaaiabigdaXiabigdaXiabdMgaPbqabaaaaaa@9739@

Here, *x*_1*i*_,*x*_2*i*_...*x*_11*i *_denote the data sets including index variables for individual years.

### Baseline

The baseline for Model 1 was defined as:

log⁡(αj)=log⁡(δ)+βsinsin⁡(2π52tj)+βcos⁡cos⁡(2π52tj)+βint⁡
 MathType@MTEF@5@5@+=feaafiart1ev1aaatCvAUfKttLearuWrP9MDH5MBPbIqV92AaeXatLxBI9gBaebbnrfifHhDYfgasaacH8akY=wiFfYdH8Gipec8Eeeu0xXdbba9frFj0=OqFfea0dXdd9vqai=hGuQ8kuc9pgc9s8qqaq=dirpe0xb9q8qiLsFr0=vr0=vr0dc8meaabaqaciaacaGaaeqabaqabeGadaaakeaacyGGSbaBcqGGVbWBcqGGNbWzcqGGOaakcqaHXoqydaWgaaWcbaGaemOAaOgabeaakiabcMcaPiabg2da9iGbcYgaSjabc+gaVjabcEgaNjabcIcaOiabes7aKjabcMcaPiabgUcaRiabek7aInaaBaaaleaacqqGZbWCcqqGPbqAcqqGUbGBaeqaaOGagi4CamNaeiyAaKMaeiOBa42aaeWaaeaadaWcaaqaaiabikdaYiabec8aWbqaaiabiwda1iabikdaYaaacqWG0baDdaWgaaWcbaGaemOAaOgabeaaaOGaayjkaiaawMcaaiabgUcaRiabek7aInaaBaaaleaacyGGJbWycqGGVbWBcqGGZbWCaeqaaOGagi4yamMaei4Ba8Maei4Cam3aaeWaaeaadaWcaaqaaiabikdaYiabec8aWbqaaiabiwda1iabikdaYaaacqWG0baDdaWgaaWcbaGaemOAaOgabeaaaOGaayjkaiaawMcaaiabgUcaRiabek7aInaaBaaaleaacyGGPbqAcqGGUbGBcqGG0baDaeqaaaaa@6D85@

Since the baseline is a mean of all years, the yearly parameter is of no importance; hence baseline for Model 1 and 2 are identical.

### Model fitting

For the models, the question of interest is if the presence of influenza affects the number of IPD cases. Our hypothesis is that a random case of IPD could occur due to a previous influenza infection. This could take place one or several weeks after the influenza infection. In order to investigate a possible delay in time in acquirement of IPD due to influenza, both models were tested in five modes: no lag, 1, 2, 3 and 4 weeks lag. For all calculations, STATA 8.0 was used. The Negative binomial regression models were calculated using function 'nbreg'. In order to establish if a negative binomial regression model should be used instead of Poisson, i.e. to establish if overdispersion was present, Pearson, χ^2 ^statistics were calculated for each of the evaluated models. See appendix B for details.

To evaluate which model was preferred, the log-likelihood was calculated for each model.

## Results

**Figure 3:**
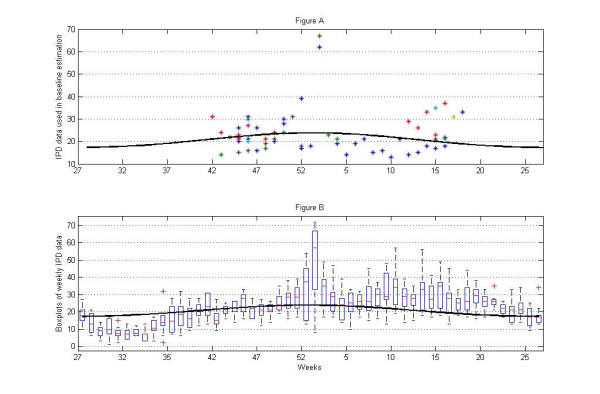
**A**. Each marker (star) represent number of IPD cases during influenza free weeks, in the time frame when influenza was monitored. Hence it shows the data points used in baseline estimation. The solid line represents baseline. **B**. For each week, the distributions of all IPD data during the period 1993–2004 are presented as box plots. The solid line represents baseline.

The number of reported influenza and IPD cases and number of laboratory confirmed domestic cases of influenza are presented in Figure 1. Number of IPD cases and the presence of influenza (indicator variable used in the calculations), over the study period is shown in Figure 2. Also, weekly distributions of number of IPD cases, presented as weekly box plots, are shown in Figure 3B. The analyses of data was made using two approaches: 1) estimating number of excess cases due to the influenza parameter in the models where presence of influenza parameter was significant, and 2) calculating mean difference of IPD cases (observed) from the baseline (expected).

**Figure 4:**
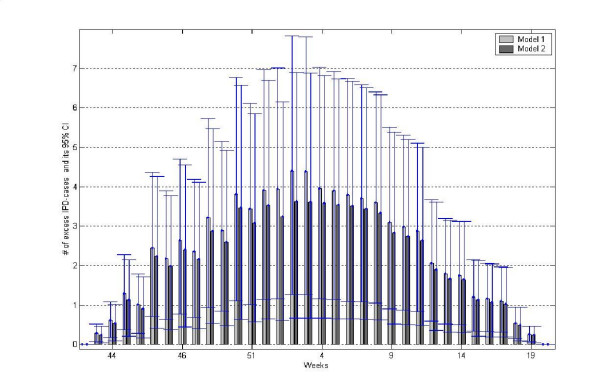
Bars represent number of weekly excess cases due to influenza parameter in Model 1 and 2, with three-week lag. Furthermore, the 95% Confidence Intervals for influenza parameter, for Model 1 and 2, are shown.

Firstly, we studied the estimated value of influenza parameter, β_inf_, its confidence intervals and p-values for both models in five modes: no lag, 1, 2, 3 and 4 weeks lag, in order to establish if the influenza indicator had an impact on the IPD data set. For both models the lag time of three and four weeks was significant, see Table 1. For model 1, also the two weeks lag seemed to be of importance. The Akaike Information Criterion estimates (AIC) were of similar value, see Table 1, hence this estimate did not clearly point out any of the models as better or worse fitted as compared to the others. To further estimate the impact of the influenza parameter, the models with significant parameter β_inf_, were recalculated without the term β_inf_. Setting the term β_inf _to zero is equivalent with calculating the model with *x*_0*i *_= 0. This could estimate number of IPD cases that should have occurred if no influenza was present or if influenza had no effect. The overall mean difference of the models and its 95% Confidence Intervals (c.i.) for each week were then calculated, and its yearly distribution is shown in Figure 4. The mean annual effect of the influenza epidemics was an excess of 81 IPD cases in Model 1 with the lag time of 3 weeks and 72 in Model 2 with the same lag (Table 2). This could translate to an attributable fraction of influenza-associated IPD 6–7% when considering the whole year, and 12–13% when only considering the seasons when influenza is monitored. Seasonal excess morbidity due to influenza parameter in those models had its peak in week 2, as shown in Figure 4. The models showed that the weekly mean number of excess cases at week 2 was 3.6 for Model 1 with 3-week lag, and 4.4 for Model 2 with 3-week lag. Pearson χ^2 ^statistics indicated that a negative binomial approach was to be preferred over a Poisson model. Here Pearson χ^2^/n statistic is estimated to 3.10 for Model 1 with 3 weeks lag and 3.19 for Model 3 with 3 weeks lag.

**Table 1:** Results of estimates for β_inf_, with its 95% Confidence Intervals (c.i.) in parenthesis. Log likelihood values for each model and lag. Log likelihood values for the null-model were -1827.19 for Model 1 and -1816.85 for Model 2. The significant estimates of β_inf _are marked with (*) and (**).

	Influenza parameter, β_inf _and its 95% c.i.	P-value	Log likelihood	AIC
Model 1				
No lag	0.05 (-0.05 – 0.15)	0.36	-1826.8	3665.5
1 week lag	0.13 (0.03 – 0.23)	0.012*	-1824.0	3660.0
2 weeks lag	0.11 (0.01 – 0.21)	0.029*	-1824.8	3661.6
3 weeks lag	0.14 (0.04 – 0.24)	0.005**	-1823.2	3658.3
4 weeks lag	0.13 (0.03 – 0.22)	0.008**	-1823.7	3659.3
Model 2				
No lag	0.03 (-0.07 – 0.14)	0.81	-1816.8	3663.6
1 week lag	0.105 (-0.001 – 0.211)	0.051	-1814.9	3659.9
2 weeks lag	0.10 (-0.01 – 0.20)	0.120	-1815.6	3661.3
3 weeks lag	0.13 (0.02 – 0.23)	0.021*	-1814.2	3658.4
4 weeks lag	0.11 (0.01 – 0.21)	0.031*	-1814.5	3659.1

**Table 2:** Yearly number of excess IPD cases, based on influenza parameters effect in the models and difference in data and baseline. Also, 95% Confidence intervals for the number of cases in from the models and Baseline are presented in parenthesis.

	Number of cases	Percent per year	Percent per season
Model 1 (3 weeks lag)	81 (24 – 243)	7% (2 – 12%)	13% (4 – 24%)
Model 2 (3 weeks lag)	72 (14 – 138)	6% (1 – 12%)	12% (2 – 23%)
Baseline (3 weeks lag)	118 (71 – 161)	10% (6 – 14%)	20% (12 – 27%)

Secondly, a baseline was created in order to establish a mean level of IPD-incidence when influenza was absent. The baseline was modeled from all influenza-free weeks when influenza was monitored (winter season) during the 11-year study period; however, during 2003 influenza occurred in all 13 studied weeks, hence, that year did not contribute to the estimation of baseline. For each year, a mean difference between the number of IPD (observed cases) and the baseline (expected cases) was calculated. We choose a 3 weeks lag in the calculations of baseline, since previous calculations (estimates of influenza as an index variable in the models) showed closest association between influenza and IPD with the lowest p-value, using this lag, see Table 1. The mean yearly distribution of data and the estimated baselines are shown in Figure 3A. 95% confidence intervals was calculated using bootstrap. The mean annual difference of IPD was 118 cases. This corresponds to 10% considering the whole year and 20% during the season of monitoring influenza.

## Discussion and conclusion

In this paper, two approaches are used to study the impact of influenza on IPD: the first one estimates impact of an influenza parameter in the models, and the second estimates the mean difference of IPD cases from an influenza-free baseline. Both methods used in this study indicated an increase in number of IPD cases due to influenza. Since both take different factors into account, it is difficult to decide which one of them should be preferred. Hence, conclusions should be made taking both methods into account.

The first approach, when the effect of the influenza parameter is estimated, uses influenza data as an indicator variable, which most likely underestimates the impact of influenza during the winter season. This could probably explain why the results in this approach give lower estimates of number of IPD cases related to influenza, than the baseline approach. Furthermore, in this method, the relevance of the influenza parameter in the model and its effect on subsequent IPD cases is studied. The result shows that the incorporation of a time lag of three to four weeks lag puts forward the significance of influenza indicator variable in both models. Also, as shown can be seen in Table 1, for both models the estimates of β_inf _increase with the size of the lag and peak at 3 weeks lag, indicating that there is a delay which must be taken into account. However, since influenza data is transformed to be an indicator variable of influenza, there can be no inference made about the relative changes in number of IPD by increasing or decreasing the number of influenza by one unit. Also, even though Model 2 did not appear to be statistically better (give better fit to the IPD data) than Model 1, we chose to include it in this report, since it is interesting that even when taking into account differences in mean values of IPD between the individual years, the results are similar.

The second approach, where a baseline is constructed, is based on a subset of the IPD data set, in other words the baseline is constructed of all weeks when influenza is not reported. The validity of the baseline varies over the season, with lowest variance in the spring and fall and largest variation during winter (since in this time of year, influenza is often present, hence, very few data points are used in the estimation). Since influenza is not monitored during summer, there is no data from this period used in the estimation of the models.

Our study estimates conservatively that 12–20% of all IPD cases (during influenza season) in Sweden are due to previous influenza. As IPD remains associated with severe morbidity and mortality even in developed countries, this study gives a clear indication of the added impact influenza has on the public health by causing severe secondary bacterial infections.

In order to detect a case of IPD in the surveillance system, a sample must be collected and sent to the laboratory for culturing. In the analysis, the date of culture was used as the onset date. Since onset of IPD disease is often of acute nature, it is realistic to assume that the culturing is made closely to the onset of disease, with no major delays between detection and report of cases.

As mentioned in previous studies [30], an increase or decrease in incidence can be observed due to altered routines in culturing. However, no active interventions or routine changes in the culturing tradition of IPD have been executed during 1994–2004.

Our results may be biased only by factors that systematically change the temporal patterns (other than seasonal, annual or long-term trends) of either influenza or IPD diagnoses. One possibility of bias is therefore changes in health-care seeking behavior and patient-sampling due to holiday periods. We have therefore studied the effects of adding a dummy variable for holiday seasons into the model, with little effect on our results or conclusions.

Also, different strains of influenza may be of varying virulence, generating an irregular amount of cases of invasive disease. A further potential limitation of this study, is that no age-stratified analysis is made, due to lack of age-specific data. Finally, since this is an epidemiological study, based on two independently collected data sets, no conclusion can be made on an individual level concerning a cause and effect of influenza.

Previous studies of the impact of influenza on pneumococcal infections have been pursued along different lines. Already during the Spanish flu pandemic of 1918 and 1919, causing 40 to 50 million lives[31], secondary pneumococcal pneumonia was noted as an often-fatal complication[32,33]. Later surveys have more in detail studied the temporal relation between the two infections[4,21-24,4,21-24]. However, none of the studies on the temporal association take into account the simultaneous seasonal occurrence of influenza and IPD, and the results may therefore be confounded by any factor, which varies by season. Talbot et al concluded in their study, a positive correlation between laboratory isolates of influenza, in adults (age >= 18 years) for 0–4 weeks lag. [25] However, since influenza is a seasonal component, it is not clear if the positive correlation is a result of direct influenza impact or other seasonal components, with the same (seasonal) frequency. In our study, we set influenza to an indicator (0/1) variable, pointing out a start and an end of an influenza season for each year. This approach allows us to study the association to IPD in a less seasonal coinciding way, since we only know if influenza is present or absent. To our knowledge, our study is the first study to closely model and quantify the association between influenza and IPD, using a model that reduces influenza to a variable indicating its presence or absence, hence, not allowing the seasonal increase of influenza to have an overall impact on the result. Our study confirms the association between the two diseases even after taking into account seasonal variation, and also shows that the strength of this association is highly seasonal with a peak excess of IPD morbidity due to influenza in January.

Finally, our findings, considering the association between influenza and IPD morbidity, coincide, despite the fact that two different approaches are used to define number of excess IPD cases. Furthermore, the detection of a lag between the influenza and IPD morbidity falls out to be an important component in forecasting amount of IPD cases, hence public health measures against influenza and IPD are preferably considered together.

## Appendix A

### Models

In the models, denote number of IPD cases as in week *t*_*i *_as *y*_*i*_. In Poisson regression models, the outcome *y*_*i *_is considered Poisson distributed with mean μ_*i*_., i.e. E[Y_i_] = μ_*i *_and variance Var(Y_i_) = μ_*i*_. Negative binomial models are equivalent to the Poisson model (where *Y*_*i *_~ *P*_*o*_(μ_*i*_)) in the estimation of mean value but allow for a greater variance (overdispersion). In these models, the assumption of equal expected value and the variance *E*[*y*_*i*_|μ_*i*_] = *Var*(*y*_*i*_|μ_*i*_) = μ_*i*_, was replaced by the assumption that the intensity parameter μ_*i *_is Gamma distributed, so that: μ_*i *_~ Γ(α_*i*_,δ). Hence, expected value of μ is: *E*[μ_*i*_] = αiδ
 MathType@MTEF@5@5@+=feaafiart1ev1aaatCvAUfKttLearuWrP9MDH5MBPbIqV92AaeXatLxBI9gBaebbnrfifHhDYfgasaacH8akY=wiFfYdH8Gipec8Eeeu0xXdbba9frFj0=OqFfea0dXdd9vqai=hGuQ8kuc9pgc9s8qqaq=dirpe0xb9q8qiLsFr0=vr0=vr0dc8meaabaqaciaacaGaaeqabaqabeGadaaakeaadaWcaaqaaiabeg7aHnaaBaaaleaacqWGPbqAaeqaaaGcbaGaeqiTdqgaaaaa@3191@ and the variance in *Var*(μ_*i*_) = αiδ2
 MathType@MTEF@5@5@+=feaafiart1ev1aaatCvAUfKttLearuWrP9MDH5MBPbIqV92AaeXatLxBI9gBaebbnrfifHhDYfgasaacH8akY=wiFfYdH8Gipec8Eeeu0xXdbba9frFj0=OqFfea0dXdd9vqai=hGuQ8kuc9pgc9s8qqaq=dirpe0xb9q8qiLsFr0=vr0=vr0dc8meaabaqaciaacaGaaeqabaqabeGadaaakeaadaWcaaqaaiabeg7aHnaaBaaaleaacqWGPbqAaeqaaaGcbaGaeqiTdq2aaWbaaSqabeaacqaIYaGmaaaaaaaa@32B0@. The expected value for *y*_*i *_is then *E*[*y*_*i*_] = *E*[*E*[*y*_*i*_|μ_*i*_]] = αiδ
 MathType@MTEF@5@5@+=feaafiart1ev1aaatCvAUfKttLearuWrP9MDH5MBPbIqV92AaeXatLxBI9gBaebbnrfifHhDYfgasaacH8akY=wiFfYdH8Gipec8Eeeu0xXdbba9frFj0=OqFfea0dXdd9vqai=hGuQ8kuc9pgc9s8qqaq=dirpe0xb9q8qiLsFr0=vr0=vr0dc8meaabaqaciaacaGaaeqabaqabeGadaaakeaadaWcaaqaaiabeg7aHnaaBaaaleaacqWGPbqAaeqaaaGcbaGaeqiTdqgaaaaa@3191@ and the variance is:

*Var*[*y*_*i*_] = *E*[*Var*[*y*_*i*_|μ_*i*_]] + *Var*[*E*[*y*_*i*_|μ_*i*_]] = αiδ(1+1δ)
 MathType@MTEF@5@5@+=feaafiart1ev1aaatCvAUfKttLearuWrP9MDH5MBPbIqV92AaeXatLxBI9gBaebbnrfifHhDYfgasaacH8akY=wiFfYdH8Gipec8Eeeu0xXdbba9frFj0=OqFfea0dXdd9vqai=hGuQ8kuc9pgc9s8qqaq=dirpe0xb9q8qiLsFr0=vr0=vr0dc8meaabaqaciaacaGaaeqabaqabeGadaaakeaadaWcaaqaaiabeg7aHnaaBaaaleaacqWGPbqAaeqaaaGcbaGaeqiTdqgaamaabmaabaGaeGymaeJaey4kaSYaaSaaaeaacqaIXaqmaeaacqaH0oazaaaacaGLOaGaayzkaaaaaa@3791@. The factor 1+1δ
 MathType@MTEF@5@5@+=feaafiart1ev1aaatCvAUfKttLearuWrP9MDH5MBPbIqV92AaeXatLxBI9gBaebbnrfifHhDYfgasaacH8akY=wiFfYdH8Gipec8Eeeu0xXdbba9frFj0=OqFfea0dXdd9vqai=hGuQ8kuc9pgc9s8qqaq=dirpe0xb9q8qiLsFr0=vr0=vr0dc8meaabaqaciaacaGaaeqabaqabeGadaaakeaacqaIXaqmcqGHRaWkdaWcaaqaaiabigdaXaqaaiabes7aKbaaaaa@3123@ measures overdispersion. [23]

Now, when Poisson or negative binomial regression is used, the logarithmic link function is applied so *E*[*y*_*i*_] = log(μ_*i*_), and the modeled response variable is:

log⁡(μi)=log⁡(αiδ)=log⁡(αi)−log⁡(δ).
 MathType@MTEF@5@5@+=feaafiart1ev1aaatCvAUfKttLearuWrP9MDH5MBPbIqV92AaeXatLxBI9gBaebbnrfifHhDYfgasaacH8akY=wiFfYdH8Gipec8Eeeu0xXdbba9frFj0=OqFfea0dXdd9vqai=hGuQ8kuc9pgc9s8qqaq=dirpe0xb9q8qiLsFr0=vr0=vr0dc8meaabaqaciaacaGaaeqabaqabeGadaaakeaacyGGSbaBcqGGVbWBcqGGNbWzcqGGOaakcqaH8oqBdaWgaaWcbaGaemyAaKgabeaakiabcMcaPiabg2da9iGbcYgaSjabc+gaVjabcEgaNnaabmaabaWaaSaaaeaacqaHXoqydaWgaaWcbaGaemyAaKgabeaaaOqaaiabes7aKbaaaiaawIcacaGLPaaacqGH9aqpcyGGSbaBcqGGVbWBcqGGNbWzcqGGOaakcqaHXoqydaWgaaWcbaGaemyAaKgabeaakiabcMcaPiabgkHiTiGbcYgaSjabc+gaVjabcEgaNjabcIcaOiabes7aKjabcMcaPiabc6caUaaa@54A1@

### Baseline

In equations below, *y*_*j*,*k *_is negative binomial distributed, *y*_*j*,*k *~ Γ_(α_*j*_,δ) where *j *indicates weeks (1 to 52) and *k *years (1 to 11).

## Appendix B

### Pearson χ^2 ^statistics

Pearsons χ^2 ^statistics is defined as: X2=∑i=1n(yi−μ^μ^)2
 MathType@MTEF@5@5@+=feaafiart1ev1aaatCvAUfKttLearuWrP9MDH5MBPbIqV92AaeXatLxBI9gBaebbnrfifHhDYfgasaacH8akY=wiFfYdH8Gipec8Eeeu0xXdbba9frFj0=OqFfea0dXdd9vqai=hGuQ8kuc9pgc9s8qqaq=dirpe0xb9q8qiLsFr0=vr0=vr0dc8meaabaqaciaacaGaaeqabaqabeGadaaakeaacqWGybawdaahaaWcbeqaaiabikdaYaaakiabg2da9maaqahabaWaaeWaaeaadaWcaaqaaiabdMha5naaBaaaleaacqWGPbqAaeqaaOGaeyOeI0IafqiVd0MbaKaaaeaacuaH8oqBgaqcaaaaaiaawIcacaGLPaaaaSqaaiabdMgaPjabg2da9iabigdaXaqaaiabd6gaUbqdcqGHris5aOWaaWbaaSqabeaacqaIYaGmaaaaaa@4153@ where *y*_*i *_is observed number of IPD cases and μ^
 MathType@MTEF@5@5@+=feaafiart1ev1aaatCvAUfKttLearuWrP9MDH5MBPbIqV92AaeXatLxBI9gBaebbnrfifHhDYfgasaacH8akY=wiFfYdH8Gipec8Eeeu0xXdbba9frFj0=OqFfea0dXdd9vqai=hGuQ8kuc9pgc9s8qqaq=dirpe0xb9q8qiLsFr0=vr0=vr0dc8meaabaqaciaacaGaaeqabaqabeGadaaakeaacuaH8oqBgaqcaaaa@2E72@ is the model estimate. If the Person χ^2 ^statistic divided with the number of observation is greater than 1, X^2^/n > 1, indicates that overdispersion is present and that the negative binomial model should be preferred over a Poisson model. In all the calculated models the result was X^2^/n > 3, hence, the negative binomial approach was used.

## Competing interests

There are no financial or other relationships that might lead to a conflict of interest. The Swedish Institute of Infectious Disease Control covered all costs related to preparing this manuscript.

## Authors' contributions

Katarzyna Grabowska carried out the statistical analysis and drafted the manuscript.

Liselotte Högberg participated in data collection, study design, carried out the epidemiological analysis and helped to draft the manuscript. Åke Svensson, Pasi Penttinen and Karl Ekdahl participated in the study design and helped to draft the manuscript. At the time of the study, co-author Karl Ekdahl was employed at the Department of Epidemiology, Swedish Institute for Infectious Disease Control.

## Pre-publication history

The pre-publication history for this paper can be accessed here:


